# Evaluation of the Vertical Accuracy of Open Global DEMs over Steep Terrain Regions Using ICESat Data: A Case Study over Hunan Province, China

**DOI:** 10.3390/s20174865

**Published:** 2020-08-28

**Authors:** Zhiwei Liu, Jianjun Zhu, Haiqiang Fu, Cui Zhou, Tingying Zuo

**Affiliations:** 1 The School of Geosciences and Info-Physics, Central South University, Changsha 410083, China; liuzhiwei@csu.edu.cn (Z.L.); haiqiangfu@csu.edu.cn (H.F.); zty_csu@csu.edu.cn (T.Z.); 2 College of Science, Central South University of Forestry and Technology, Changsha 410001, China; cuizhou@csuft.edu.cn

**Keywords:** DEM, SRTM, ASTER GDEM, AWED30 DEM, TanDEM-X DEM, ICESat-2, vertical error, accuracy

## Abstract

The global digital elevation model (DEM) is important for various scientific applications. With the recently released TanDEM-X 90-m DEM and AW3D30 version 2.2, the open global or near-global coverage DEM datasets have been further expanded. However, the quality of these DEMs has not yet been fully characterized, especially in the application for regional scale studies. In this study, we assess the quality of five freely available global DEM datasets (SRTM-1 DEM, SRTM-3 DEM, ASTER GDEM2, AW3D30 DEM and TanDEM-X 90-m DEM) and one 30-m resampled TanDEM-X DEM (hereafter called TDX30) over the south-central Chinese province of Hunan. Then, the newly-released high precision ICESat-2 (Ice, Cloud, and land Elevation Satellite-2) altimetry points are introduced to evaluate the accuracy of these DEMs. Results show that the SRTM1 DEM offers the best quality with a Root Mean Square Error (RMSE) of 8.0 m, and ASTER GDEM2 has the worst quality with the RMSE of 10.1 m. We also compared the vertical accuracies of these DEMs with respect to different terrain morphological characteristics (e.g., elevation, slope and aspect) and land cover types. It reveals that the DEM accuracy decreases when the terrain elevation and slope value increase, whereas no relationship was found between DEM error and terrain aspect. Furthermore, the results show that the accuracy increases as the land cover type changes from vegetated to non-vegetated. Overall, the SRTM1 DEM, with high spatial resolution and high vertical accuracy, is currently the most promising dataset among these DEMs and it could, therefore, be utilized for the studies and applications requiring accurate DEMs.

## 1. Introduction

Digital elevation model (DEM), containing the basic information about the topographic features representing the actual Earth’s surface [1]. It has been used for various fields, such as the glacier mass estimation [2], forest inventory [3,4], geological hazard monitoring [5] and natural hazard mapping [6], and its accuracy determines the reliability of these results.

In the past decades, the development of Earth observation technologies brought a variety of global or near-global scale digital elevation models (DEMs), including SRTM DEM [7], ASTER GDEM2 [8,9], AW3D30 DEM [10] and TanDEM-X DEM [11,12,13]. ASTER GDEM2 and AW3D30 DEM were obtained by photogrammetric methods using stereo data, but SRTM DEM and TanDEM-X DEM were generated by interferometric synthetic aperture radar (InSAR). Due to different imaging configurations and data processing methods, these DEM products contain various errors [14]. In addition, compared with national DEM products, these global DEMs have lower vertical accuracy. However, for regional scale geographical studies, it is difficult to obtain highly accurate national DEMs covering the entire region, the freely available medium-resolution global DEMs are thus often used instead. Therefore, evaluating the accuracy of these global DEMs is necessary.

To reveal the vertical accuracy of different DEM datasets, many studies compared the DEM values against highly accurate ground points (e.g., GPS measurement, NASA ICESat/GLAS) [15,16,17,18,19,20,21,22,23,24,25,26] or reference DEMs [27,28,29]. Furthermore, independent studies on the global or regional DEMs show that the real vertical accuracy is often higher than the official accuracy [24]. However, comprehensive vertical accuracy evaluations are scarce for the DEMs over the south-central and southwestern regions of China [23,29], especially Hunan province, where the topography and land cover types are complex. In addition, the ICESat/GLAS data is often selected as the reference data to estimate the DEM accuracy, due to its high accuracy and wide coverage [15,16,17]. However, the accuracy of ICESat/GLAS data with larger-footprint size are highly susceptible to the influence of ground slope. Thus, it is not always appropriate to verify the accuracy of DEM, especially in rugged areas. In this paper, we selected the ICESat-2 altimetry data to evaluate the vertical accuracy of different DEM datasets, which include three with 30-m cell resolution (SRTM1, ASTER GDEM2 and AW3D30), one 30-m resampled TanDEM-X DEM (hereafter called TDX30), and two with 90-m resolution (SRTM3 and TanDEM-X 90m DEM (TDX90)).

The structure of this paper is outlined as follows: Section 2 briefs the study site and data sources. Then, the assessment method is introduced in Section 3. The overall performances of these DEMs are assessed in terms of vertical accuracy using highly accurate ICESat-2 altimetry points in Section 4. In that section, the performances of each DEM with different topographic characteristics (such as the terrain elevation, terrain slope and aspect) and land cover types are tested in the selected study area. Furthermore, the impacts of different reference data on the DEM selection are analyzed. Finally, some conclusions are drawn in Section 6.

## 2. Study Area and Data

### 2.1. Study Area

**Figure 1:**
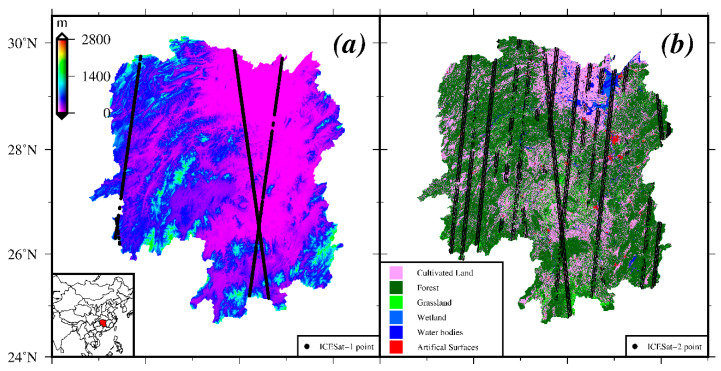
(**a**) Digital elevation map and the filtered footprints of Ice, Cloud, and land Elevation Satellite-1 (ICESat-1) reference data points (black points). (**b**) The map of land cover types and the footprints of the selected Ice, Cloud, and land Elevation Satellite-2 (ICESat-2) altimetry data (black points).

The study was carried in south-central Chinese province in Hunan, which is characterized by hilly topography with the elevation ranging from 0 m to 2100 m above the WGS84 ellipsoid, and bounded between 24°38′ N and 30°08′ N latitudes and 108°47′ E and 114°15′ E longitudes. The total area of Hunan province is approximately 2,118,000 km^2^. Figure 1a,b display the corresponding topography and land cover map over the study area, respectively. In addition, this study area contains a highly diverse land cover types and ecosystems due to the complex terrains and weather. Under the continental subtropical monsoon climate, dense vegetation significantly decreases the DEM accuracy [3,30].

### 2.2. Study Datasets

#### 2.2.1. SRTM DEM

Shuttle Radar Topography Mission (SRTM) is a project jointly carried out by the National Aeronautics and Space Administration (NASA) and the National Imagery and Mapping Agency (NIMA) of the U.S. Department of Defense. Its main objective was to produce a DEM of the Earth’s land surface between 60° N and 56° S [31]. The data were acquired from the 11th to the 22nd of February 2000, by two InSAR sensors, the C-band radar system (SIR-C) and the X-band system (X-SAR). The SRTM X-band has a small swath width that leads to small coverage [3]. This study assesses the C-band SRTM DEM, which is better known and more widely available.

The first version (1″ resolution and 3″ resolution) of the C-band SRTM data was provided in 2003. The 1″ resolution data (approximately 30 m at the equator) were available only for the United States, and the 3″ resolution data (approximately 90 m at the equator) data were sampled for the rest of the world. On 23 September 2014, the 1″ global SRTM-1 V3 DEM product that is known as “SRTM plus” or “SRTM NASA Version 3” became freely available for the regions outside the United States. It provides the worldwide coverage of void-filled elevation data using extra auxiliary DEM datasets. Version 4.1 is the highest accuracy dataset of the SRTM, at 3″ resolution, which was released by the Consultative Group for International Agriculture Research Consortium for Spatial Information (CGIAR-CSI). Different from Version 3, Version 4.1 uses several interpolation techniques [32] and extra auxiliary DEMs to fill the voids, and uses SRTM-30 for large voids [33]. Considering the consistency of geolocation [34], the SRTM-1 and SRTM-3 of Version 3 were selected and downloaded from the United States Geological Survey (USGS) Earth Explorer site (https://earthexplorer.usgs.gov/) for this study.

#### 2.2.2. ASTER GDEM

The ASTER instrument was built by Ministry of Economy, Trade and Industry (METI) of Japan and launched onboard NASA’s Terra spacecraft in December 1999. Its nadir-infrared subsystem contains nadir-viewing and backward-viewing telescopes, which together enable it to acquire along-track stereo image data. The spatial resolution is 15 m in the horizontal plane. About 1,500,000 scenes of stereoscopic data were acquired during its operation.

These images were processed by a fully-automated processing chain to generate the ASTER GDEM version 1 (ASTER GDEM1) in 2009. This product covers about 99% of the land surface between 83° N to 83° S, and its horizontal resolution is around 30 m at the equator. However, even though the ASTER DEM product at this version used all available original scenes, it still contains artifacts and outliers. The improved ASTER GDEM V2 released in 2011 is an improved ASTER GDEM. It was generated from more ASTER stereo pairs by an improved processing algorithm. ASTER GDEM V2 downloaded from the USGS Earth Explorer site was used in this study.

#### 2.2.3. ALOS WORLD 3D 30 m (AW3D30) DEM

The Advanced Land Observing Satellite (ALOS) was developed by JAXA (Japan Aerospace Exploration Agency, Tokyo, Japan), and launched on 24 January 2006. ALOS was developed to contribute to the fields of mapping, precise regional land coverage observation, disaster monitoring, and resource surveying. The Panchromatic Remote-sensing Instrument for Stereo Mapping (PRISM) sensors was an optical instrument onboard the ALOS and it was in operation between 2006 and 2011, which consisted of three along-track panchromatic radiometers at nadir (NDR), forwards (FWD) and backwards (BWD), and has a 2.5 m spatial resolution. From the 2.5-m ALOS along-track stereo observation images, a global DEM/DSM dataset named Advanced World 3D (AW3D) was generated with a 5 m grid spacing [35,36]. The 5 m resolution AW3D dataset is distributed commercially, but the 30 m resolution DEM dataset AW3D30 is freely available. Since its release, the quality of AW3D has been progressively improved.

In March 2017, Japan Aerospace Exploration Agency (JAXA) released the AW3D30 DEM Version 1.1. It has filled the void pixels in version 1.0, which are caused by the cloud and snow in the area between 60° N and 60° S. Version 2.1, released in April 2018, has removed the absolute offset errors and relative striping errors along satellite orbits in the area between 60° N and 60° S and part of the arctic in the first version. The latest version, version 2.2, was made available in April 2019, which has improvements over the old versions, especially for the northern region over 60° N. This version adds the missing tiles over the land of no-data or low-quality areas, and updates the coastline. We downloaded the AW3D30 version 2.2 from the Earth Observation Research Center JAXA (https://www.eorc.jaxa.jp/ALOS/en/aw3d30/data/index.htm).

#### 2.2.4. TanDEM-X 90 m DEM (TDX90)

The TerraSAR-X add-on for Digital Elevation Measurements (TanDEM-X) is an earth observation radar mission to acquire a precise 3D global Earth’s land surface elevation dataset with homogeneous quality and unprecedented accuracy [12,37]. This goal was achieved by extending the TerraSAR-X (TSX) mission by a second satellite of TanDEM-X (TDX), which flew in twin formation with baseline distances of typically 300–500 m. Thus bi-static interferometry is realized by transmitting pulses from the antenna of one of the satellites and receiving the backscattered signals simultaneously from two satellites. This reduces or removes the impacts of significant temporal decorrelation and atmospheric disturbances of the traditional repeat-pass InSAR on the acquisition of highly accurate cross-track interferograms [11,38].

The twin-satellite constellation TSX and TDX acquired the data for the global DEM from December 2010 to January 2015, and the finalization of the global DEM dataset is in September 2016. In 2018, the TanDEM-X 90-m DEM was released by DLR (Deutsches Zentrum für Luft-und Raumfahrt) to the scientific community and it is now freely available. TanDEM-X 90-m DEM was derived from the global DEM with a 0.4 Arc seconds posting (approximately 12 m at the equator) [39,40]. The DEM product with 12-m spatial resolution is not free of charge; thus, we assessed the freely available TanDEM-X 90-m DEM product in this paper, which was downloaded from the website provided by German Aerospace Center (DLR) (https://geoservice.dlr.de/web/dataguide/tdm90/).

#### 2.2.5. ICESat-2 Altimetry Data

The Ice, Cloud, and land Elevation Satellite (ICESat) altimetry dataset has been widely used to validate the quality of DEM, due to its very high altimetry accuracy [14,15,16,41]. The Geoscience Laser Altimeter System (GLAS) is the first space-based laser altimetry instrument onboard ICESat, which was launched on 12 January 2003. During its seven of years in-orbit operation, it completed 18 laser-operations campaigns. GLAS provided invaluable global data for estimating the Earth’s elevation with a footprint size of approximately 70 m and the along-track sampling interval of about 170 m [42,43]. More information regarding the ICESat mission can be found at the NASA website [43,44].

However, a single beam laser cannot separate surface slope effects from true elevation changes orbit by orbit bias and thus many years of data were needed to separate these two effects. In addition, the local surface slope has stronger effect on the estimation accuracy, as the accuracy drops quickly in steep areas [45]. In order to solve this problem, a new NASA space-based laser altimetry mission, ICESat-2 was launched on 15 September 2018. As a more advanced space-based Light Detection and Ranging (LiDAR), ICESat-2 has multi-beam instrument design (six beams arranged as three pairs of beams), small footprint size, and the ability to resolve rougher terrains, which can realize higher accuracy mountain measurements. Each beam pair of ICESat-2 consists of a strong and weak energy beam (4:1 ratio) which allows for local slope determination between each beam pair, as well as to compensate for varying surface reflectance. Furthermore, the strong energy beams represent the best option for detecting ground and canopy photons [46].

Based on the distribution of signal photons, the ATL08 algorithm can estimate the ground surface and top of canopy surface elevations reported on the ATL08 data product [46]. As a result, unlike ICESat/GLAS data, ICESat-2 data record ground surface elevation and the canopy height information, providing more intensive and sufficient reference data for the quality analysis of different DEMs. In the previous research, Neuenschwander et al. presented the quantitative assessment of the terrain height of ICESat-2 ATL-08 product as compared to airborne lidar data [47]. The initial result of the best fit terrain height validation over Finland indicates that the terrain residual has a mean absolute error of 0.5 m, while the Root Mean Square Error (RMSE) is 0.82 m. Thus, it is suitable to be used to assess the DEM accuracy. In this study, the altimetry data acquired by ICESat-2 in the first few months were selected to further assess the vertical accuracies of different DEMs, to help us understand the DEM accuracy and the corresponding error characteristics. In this study, the ICESat-2 ATL08 data from the NASA Earthdata Search website (https://search.earthdata.nasa.gov/search) were used for the analysis. In addition, to solidify the analysis result, ICESat-1 GLA14 version 34 was also selected as the reference data.

## 3. Method

### 3.1. Data Post-Processing

In order to assess the vertical error, the most important steps during post-processing are

(1) Merge and crop DEM, and then extract ICESat/GLAS and ICESat-2 altimetry points. The vertical and horizontal datum of all the datasets are given in Table A1, as shown in Appendix A. The WGS84 reference system and vertical datum are selected in this study.
(2) Filter outliers for ground points. Select high precision ICESat/GLAS altimetry points according to the four flags mentioned in [20] and a 50-m elevation difference threshold. A total of 95,381 filtered ICESat/GLAS elevation points are acquired (Figure 1a). Due to the lower noise of the night collections, we selected the strong beam data acquired at night to ensure the quality of ICESat-2 elevation [46,47,48]. A slope corrected best-fit terrain height (h_te_Bestfit) on the ATL08 data product was selected as the reference data, which represents the ground surface elevation. Furthermore, the flag of “*cloud_flag_atm*” was used to reduce the aerosols or clouds impacts of the acquisition. After the above processing, we collected a total of 49,707 ICESat-2 reference data points, and the corresponding footprints are shown in Figure 1b.
(3) Transform orthometric height to ellipsoidal height.

For SRTM1/3 DEM, ASTER GDEM2 and AW3D30 DEM, the global geoid model provided by National Geospatial-Intelligence Agency (NGA) was used to evaluate the geoid undulation (N), which means the distance from a point on the geoid (e.g., EGM-96) along the normal line to the ellipsoid (e.g., WGS84) [27,28] and thus the ellipsoidal height can be calculated by Equation (1):

(1) h=H+N

where h is the ellipsoidal height relative to the WGS84 ellipsoid, and H is the orthometric height with respect to EGM-96 geoid.

The height of the ICESat/GLAS data is the ellipsoidal height with respect to the TOPEX/Poseidon (T/P) ellipsoid. Although the Geographic datum between ICESat/GLAS elevation and each DEM data is different, the horizontal displacements between T/P and WGS84 ellipsoid are negligible [19]. So only the transformation of vertical datum needs to be considered and the ellipsoidal height above WGS84 can be calculated by Equation (2):

(2) $$h_{WGS84}=h_{T/P}-0.707\;m$$

where $h_{WGS84}$ is the ellipsoidal height with reference to WGS84 ellipsoid; $h_{T/P}$ is the ellipsoidal height relative to the T/P reference system; and the constant value 0.707 m means the height difference between WGS84 and T/P ellipsoids.

### 3.2. Elevation Accuracy Assessment

The elevation accuracies of these DEMs were estimated by comparing with the high qualityICESat-2 altimetry data. Because the ICESat-2 reference data are a series of discrete points, the inverse distance weighting (IDW) interpolation method [49,50] was selected to calculate the DEM elevation of each ICESat-2 data footprint. Formally, the IDW method is used to estimate the unknow value $\hat{y}\left(S_{0}\right)$ in location $S_{0}$, given the observed *y* values at sampled locations $S_{k}$ in the following manner:

(3) $$\hat{y}\left(S_{0}\right)=\sum_{k=1}^{n}\lambda_{k}y\left(S_{k}\right)$$

where the weight value $\lambda_{k}$ is calculated by:

(4) $$\lambda_{k}=d_{0k}/\sum_{k=1}^{n}d_{0k}\;\mathrm{with}\;\sum_{k=1}^{n}\lambda_{k}=1$$

Then, the elevation differences were calculated by subtracting the DEM elevation from the ICESat-2. The Mean Error (ME), Root Mean Square Error (RMSE) and Standard Deviation (STD) were calculated as follows:

(5) $$ME=\frac{1}{n}\sum_{i=1}^{n}\left(x_{i}-y_{i}\right)$$

(6) $$RMSE=\sqrt{\frac{\sum_{i=1}^{n}{\left(x_{i}-y_{i}\right)}^{2}}{n}}$$

(7) $$STD=\sqrt{\frac{\sum_{i=1}^{n}{\left[\left(x_{i}-y_{i}\right)-\bar{\left(x_{i}-y_{i}\right)}\right]}^{2}}{n-1}}$$

where n is the number of reference data points; x is the reference elevations, while y is the elevation values of DEM. The unit of the elevation data is meter.

## 4. DEM Validation Based on ICESat-2 Data

### 4.1. DEM Error Assessment

**Figure 2:**
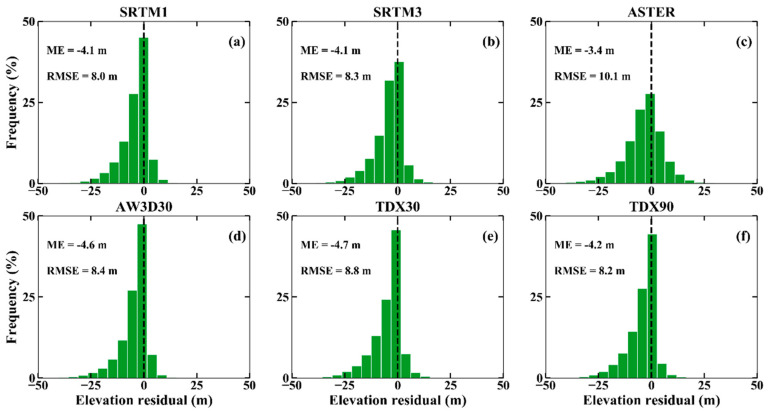
Histogram plot of the elevation errors (ICESat-2 minus different digital elevation models (DEMs)) for (**a**) SRTM1; (**b**) SRTM3; (**c**) ASTER; (**d**) AW3D30; (**e**) TDX30 and (**f**) TDX90. (TDX30: 30-m TDX DEM, TDX90: 90-m TDX DEM).

Besides the five free global DEMs, TDX30 resampled from the 90 m resolution TanDEM-X DEM by the bicubic interpolation method was also used for the error assessment. The histogram of the height differences between these DEMs and the high-precision ICESat-2 altimetry points are displayed in Figure 2. The ME values vary from −3.4 m to −4.7 m, indicating that all these DEMs provide the elevations higher than those of reference points. Furthermore, these negative ME values can be partly attributed to the impact of dense vegetation canopy, in which the short wavelength SAR or optical satellite sensors cannot ‘see’ the sub-canopy topography. In addition, we found that the errors of all DEMs are normally distributed with small negative biases and negative skewness values (see Figure 2). The RMSE values range from 8.0 m to 10.1 m. For the entire area, SRTM1 DEM shows the highest accuracy with the mean value of −4.1 m and the lowest RMSE of 8.0 m, and ASTER GDEM has the lowest quality with the highest mean value (−3.4 m) and the highest RMSE (10.1 m).

For the DEMs with the resolution of ~90 m, TDX90 DEM shows better quality than SRTM3 DEM. However, due to the stronger penetrability of C-band than that of X-band, the phase centers of SRTM DEM should be closer to the earth surface than that of TDX90 DEM. In theory, SRTM3 DEM has higher quality than TDX90 DEM, but the result is opposite in this study. This result can be partly explained by that the combination of multi-baselines or ascending and descending viewing geometry can improve the DEM quality [13]. Of course, we cannot exclude the influence of vegetation change in height over a period of more than a decade on the results obtained. For example, the natural growth or deforestation of the vegetation can result in the variation of DEM elevation, which will impact the analysis results. Future studies and analysis should answer this question.

Among the DEMs with the resolution of ~30 m, SRTM1 DEM has the best quality with ME of −4.1 m and RMSE of 8.0 m. ASTER GDEM2, AW3D30 DEM and TDX30 DEM even show lower qualities than the SRTM3 and TDX90 DEMs. This result could be partly attributed to the advantages of InSAR technology over vegetation coverage areas. In addition, the SRTM3 DEM has lower quality than SRTM1 DEM, indicating that down-sampling SRTM does reduce DEM quality, especially for mountainous topography. This also demonstrates the advantages of higher resolution DEM in depicting terrain details. TDX30 DEM has lower quality than TDX90 DEM. This result could have resulted from many reasons: (1) up-sampling TDX90 DEM may change the spatial feature information of the original DEM product. (2) the up-sampling (from TDX90 to TDX30) and IDW interpolation (from TDX30 to ICESat-2 footprint) operations were performed to assess the TDX30 DEM accuracy respectively, which will lead to the accumulation of DEM errors. Therefore, the spatial interpolation technique has no ability to improve the DEM accuracy, especially in the mountain area, as it cannot add any more topographic detail information.

TanDEM-X DEM is expected to be a new standard in global DEMs, regarding the geometric resolution, accuracy and ability to depict complex topography [11,13]. However, the TDX90 DEM is not accurate enough due to its low resolution. This conclusion is in agreement with the results of [21].

### 4.2. Influence of Elevation on the DEM Error

**Figure 3:**
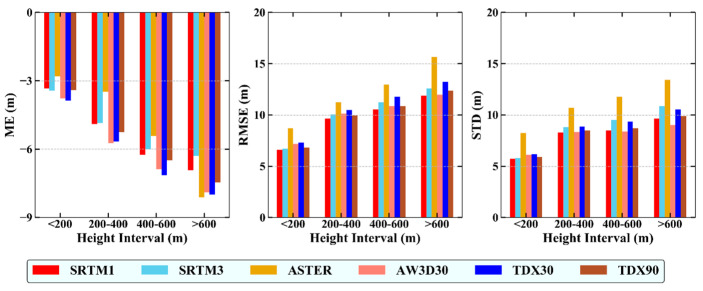
Mean Error (ME), Root Mean Square Error (RMSE) and Standard Deviation (STD) of the DEM errors.

To analyze the relationship between elevation and DEM error, the DEM errors were divided into four groups according the elevation: 0–200 m, 200–400 m, 400–600 m and >600 m. Figure 3 shows the ME, RMSE and STD values of the DEMs in different elevation ranges. The ME values exhibit general upward trends when the terrain height increases. The RMSE and STD values of the DEMs, except AW3D30, increase with the elevation. This can be explained by: (1) with the elevation increase, the terrain becomes rougher, which makes the optical photogrammetry and InSAR techniques difficult to measure the ground elevation; (2) the ground cover of higher area is dominated by forest (Figure 1), which prohibits the optical photogrammetry and short wavelength SAR (e.g., X- and C-band) from measuring the sub-canopy ground elevation. In particularly, a significant increase of RMSEs and STDs is observed when the terrain height is under 400 m (except ASTER GDEM), but the error increases slightly when the height is >400 m. The AW3D30 DEM shows immunity to the elevation change. In addition, the DEM errors with 90 m resolution increase more significantly with elevation than those of DEM at 30 m resolution, indicating that DEM with higher resolution has better tolerance to the elevation change.

### 4.3. Impact of Slope and Aspect on the DEM Error

**Figure 4:**
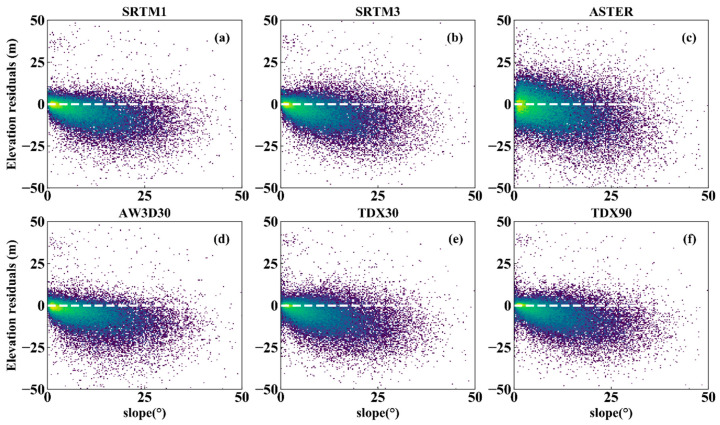
Scatter density plots between DEM error and slope for (**a**) SRTM1; (**b**) SRTM3; (**c**) ASTER; (**d**) AW3D30; (**e**) TDX30 and (**f**) TDX90.

Slope and aspect are the two main factors affecting the DEM error, since they induce geometric distortion in the imaging processing associated with optical photogrammetry and InSAR, which prohibits us from extracting true topography [51,52]. The slope and aspect at each ICESat point were calculated from the AW3D30 DEM. As Figure 4 shows, the DEM errors of all datasets decrease significantly with the increasing slope, suggesting that the terrain slope has stronger effect on the DEM errors of both InSAR and optical photogrammetry technology. Furthermore, this result provides a possibility for us to correct DEM errors related to ground slope, as demonstrated in [53].

**Figure 5:**
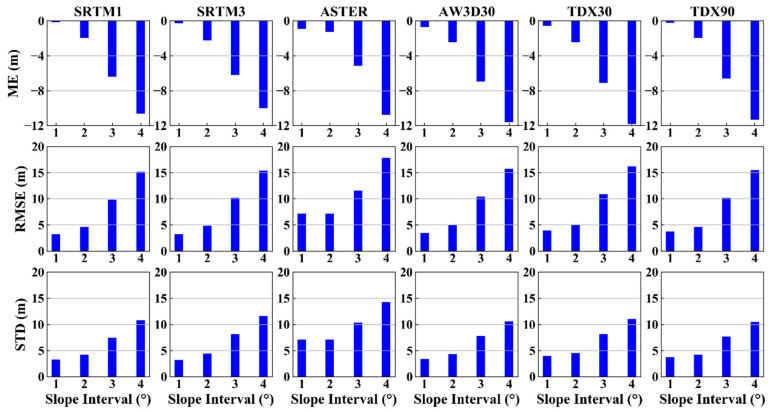
DEM error statistics of different slopes; ME (top), RMSE (middle) and STD (bottom). (1: 0–2°; 2: (2–6°]; 3: (6–25°]; 4: >25°).

**Figure 6:**
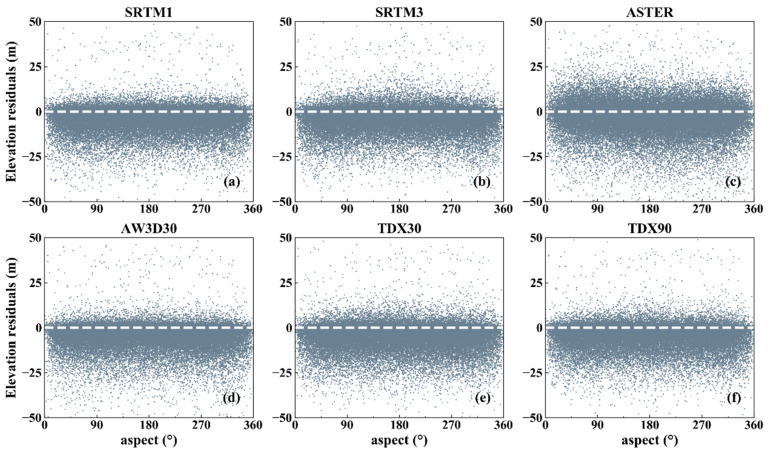
The scatter plots between DEM error and terrain aspect for (**a**) SRTM1; (**b**) SRTM3; (**c**) ASTER; (**d**) AW3D30; (**e**) TDX30 and (**f**) TDX90.

To quantify the impact of slope on DEM error, all the DEM errors were divided into four groups according to the terrain slope, which are the flat (slope angle <2°), hilly (2–6°), mountain (6–25°) and high mountain areas (>25°) [54]. The ME, RMSE and STD were then calculated and are shown in Figure 5. The MEs exhibit obvious increasing trends with the increasing terrain slope. In the areas with the slope angle smaller than 2°, the MEs of InSAR DEM (except for TDX30) are close to 0. The maximum of the MEs appear at the slope angles of >25°. In the middle and bottom panels, the RMSEs and STDs linearly increase with the slopes. In addition, the RMSE values of all datasets except for ASTER GDEM2 are less than 5 m in the region where the slope angle is under 2°. The values peak (15 m) in the areas where the slope angles are larger than 25°. These demonstrate that the terrain slope has a serious effect on the DEM errors of both InSAR and optical photogrammetry technology. Such change pattern can be explained by the influence of slope on image acquisition. In addition, when the slope is small (<25°), the RMSEs of SRTM1 and SRTM3 are similar, but when the terrain slope is >25°, the difference of their RMSEs are remarkable. This confirms that the DEMs with high resolution can depict the terrain slope. The comparison between TDX30 and TDX90 shows that the spatial interpolation cannot reduce the DEM error significantly. Thus, for flat terrains, all the DEMs excepting the ASTER GDEM2 can be used as high precision DEMs. However, for high mountain areas, SRTM1 is a better choice than other DEMs, due to its high resolution, though TDX90 shows an equal vertical accuracy with it. No clear relationship has been found between the elevation residuals and terrain aspect (Figure 6). However, some relevant researches indicate that there is an obvious cosine relationship between the DEM errors and terrain aspect [28,55], which can be attributed to the horizontal shifts between different datasets rather than the effect of terrain aspect on DEM accuracy. In order to solve this problem, co-registration of different datasets is an effective and applicable method [55,56].

### 4.4. Vertical Accuracy Versus Land Cover

Covering various regions of Hunan province, these datasets allow further study of the relationship between the DEM accuracy and terrain type and ground cover. The global land-cover model referenced in this study is the GlobaLand30 dataset produced by National Geomatics Center of China (NGCC), which represents one of the first efforts in mapping a global land cover at 30 m spatial resolution [57]. GlobaLand30 defines ten land cover categories with an overall classification accuracy of 83.51%, which has been validated [58]. In this study area, six land cover types were identified according to the distribution of ICESat-2 reference data, which are cultivated land, forest, grassland, wetland, water body, as well as artificial surfaces. We assumed that the land cover types do not change significantly during the image acquisition.

**Figure 7:**
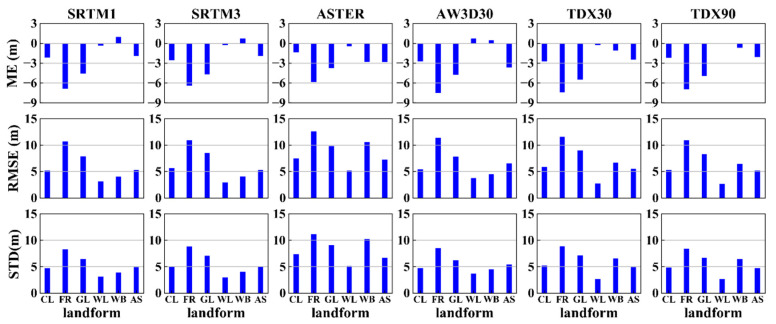
The error statistics in different land cover classes: ME (top), RMSE (middle) and STD (bottom) values of different DEMs. CL: cultivated land, FR: forest, GL: grassland, WL: wetland, WB: water body, AS: artificial surfaces.

Figure 7 shows the error statistics from all DEMs over different land cover types. The ME values (except water body of SRTM1, SRTM3 DEM and AW3D30 DEM, and the wetland of AW3D30 DEM) are negative for land cover types among all DEMs, and that of forest is higher than that of other classes. This result is related to the terrain characteristics of different land cover types. However, the variation of the MEs of the water body is related to the fluctuations of water levels. As the RMSE shows, the forest has the poorest accuracy, followed by grassland, and the cultivated land, wetland and water bodies. Artificial surfaces have the highest accuracies. Hence, the DEM accuracy increases as the land cover changes from fully vegetated to non-vegetated areas.

For DEM at 30 m resolution, SRTM1 offers the best quality in each land cover class. As AW3D30 has been calibrated based on ICESat/GLAS data, it shows a better quality than ASTER GDEM2 and TDX30 DEM. Furthermore, we can find that AW3D30 DEM shows a better accuracy over the forest area and a lower vertical accuracy than that of SRTM1 DEM over the wetland, this phenomenon can be attributed to the fitting method used for AW3D30 calibration, which raises the terrain height in lower areas (e.g., wetland) in the removal of the vegetation height signals (forest). In addition, both the ASTER and AW3D30 show lower quality than that of InSAR DEM over the region of artificial surfaces. The accuracies of the 90-m DEMs (both SRTM3 and TDX90) are nearly the same for all land cover types, except for the water body. SRTM1 performs a little better than 90 m SRTM3, because of its higher resolution. However, the resolution seems have no influence on the accuracy of TDX30 and TDX90. According the result, the following conclusion is drawn, in the case of the same land cover type, the resolution has little influence on the DEM accuracy. Finally, STD in the lower panel of Figure 7 shows similar trends as RMSE.

## 5. Discussion

### 5.1. Impacts of the Reference Data for DEM Selection

**Figure 8:**
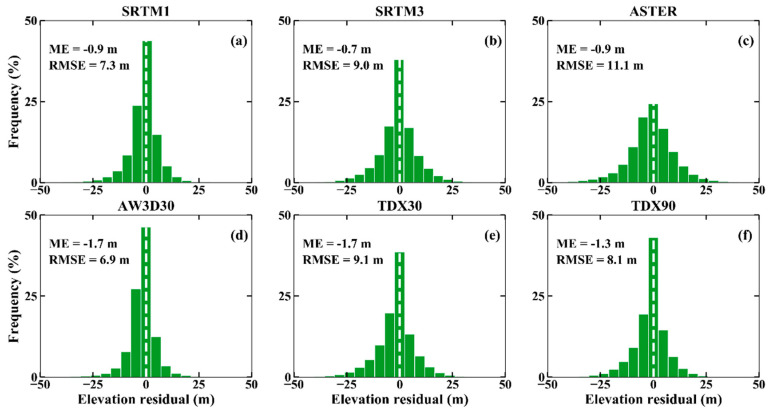
Histogram plot of elevation errors for (**a**) SRTM1; (**b**) SRTM3; (**c**) ASTER; (**d**) AW3D30; (**e**) TDX30 and (**f**) TDX90.In addition, ASTER GDEM2 (~30 m spatial resolution) has the lowest accuracy among the six DEMs, which is in agreement with the results of Figure 2 and the study of [17]. AW3D30 DEM shows the best quality (RMSE = 6.9 m), followed by SRTM1 DEM, which conflicts the results shown in Section 4.1 and the results of Maria et al. [24]. This is because AW3D30 DEM has been calibrated by the ICESat/GLAS altimetry data. When it is validated using the ICESat/GLAS data, the result will show a better but not true vertical accuracy. As a consequence, SRTM1 DEM should be recommended for the applications requiring accurate Earth surface description, as it has the best accuracy among the freely available medium-resolution DEMs. In addition, the newly released ICESat-2 is suggested as the reference height data for the DEM vertical accuracy assessment, due to its high accuracy and accessibility.

The ICESat/GLAS data were considered as the optimal choice for DEM accuracy assessment, due to its high accuracy and wide coverage [15,16,17,20,21]. They have been used by DEM publishers for DEM error calibration [59], which might have an impact on the understanding of different DEM quality. The latest or upcoming release of more accurate altimetry data (e.g., ICESat-2, GEDI) [60], however, helps us to understand the DEM errors better. In here, we also selected corresponding ICESat/GLAS elevation points over the study area to analyze the impact of different reference data on DEM selection. Then, the DEM accuracy assessment method described in Section 4.1 was used to evaluate the overall accuracy of different DEMs (Figure 8). A comparison of the results is displayed in Figure 2, where the MEs of all datasets are negative, but the absolute MEs of DEMs are smaller than that of ICESat-2. This result can be attributed to that the ICESat/GLAS data contain the ground object height information (e.g., vegetation), but ICESat-2 measures the height close to the bare-earth surface elevations.

### 5.2. Accuracy Verification Results with Respect to ICESat-2

The assessments of the vertical accuracy of the six DEMs using high-precision ICESat-2 altimetry points show that SRTM1 is significantly better than other DEMs (Figure 2), while the statistics accuracy of the AW3D30 DEM is closest to that of SRTM1 DEM, and the result is in agreement with other studies [24]. In addition, the 30 m DEMs, except ASTER GDEM2, have higher accuracies than that of the 90 m DEMs, which could be attributed to the higher spatial resolution.

As described in Section 4.2, a close relationship between DEM errors and terrain height was found (Figure 3), as has been suggested by [17] and [27]. This is due to the fact that the DEM surface is more erroneous in high-altitude areas with rugged terrains. Furthermore, the RMSE shows an increasing trend with the growing height, indicating that relief increases the uncertainty of height measurement. According the performances of different DEMs, the SRTM1 DEM is suggested to apply when the terrain height is <400 m, but when the height goes beyond 400 m, AW3D30 DEM is a suitable candidate data.

The comparison between height accuracy and terrain slope angle shows that the larger the slope inclination angle, the lower the vertical accuracy (Figure 5), which has proven in previous studies [16,17,24]. The assessment results also show that the accuracies of DEMs are less than 2 m in flat areas (slope angle less than 2°). Furthermore, the InSAR DEMs have better quality than optical DEMs in flat areas. However, the errors of all the six DEMs increase rapidly when the slope value is >2°, which is consistent with the results in [27]. Thus, as the areas where the slope is <6°, InSAR DEM should be selected and utilized. However, in concordance with Kolecka el al. [61], we did not find a clear relationship between DEM errors and terrain aspect (Figure 6). Other authors, however (e.g., [55]), did find a statistical correlation, which can be attributed by the horizontal shifts between different datasets rather than the influence of the terrain aspect on DEM accuracy.

In addition, the vertical accuracy of these DEMs across different land cover types were assessed in Section 4.4. The assessment results show that the areas with ground features have higher elevation residuals (e.g., forest and grassland), and the InSAR DEMs show better quality in the areas with artificial surface than the optical DEM. Similarly, Rexer et al. [62] and Varga et al. [63] also found that both SRTM (90 m and 30 m variants) and ASTER GDEM2 did show higher errors in areas with forest canopy cover. Thus, in the future work, how to accurately rectify the DEM deviation caused by forest canopy height is an important study to obtain the “bare-earth” elevation [3,64,65].

## 6. Conclusions

A first assessment of the freely available DEMs over the south-central Chinese province of Hunan was presented through a comparison with the highly accurate ICESat-2 altimetry points. Meanwhile, the effects of the terrain morphological characteristics (e.g., elevation, slope and aspect) and land cover type were also analyzed in the presented study.

The assessment result shows that, among the 30 m models, SRTM1 DEM performed best, followed by AW3D30 DEM and ASTER GDEM2 performed worst. SRTM1 DEM has been extensively used, and provided a good representation of the topography. AW3D30 DEM, however, shows a very uneven quality and can be used in the areas without SRTM1 DEM. It is noted that the selection of reference data can impact the analysis results as described in Section 5.1. Therefore, high-precision and independent reference data is important to assess the DEM accuracy. Among the 90 m models, the recently released TDX90 DEM shows higher quality than the SRTM3 DEM, which confirms the advantages of fusing multi-baseline or ascending and descending viewing geometry for producing InSAR DEM. However, the influence of vegetation change in height over a period of more than a decade still is not excluded.

Results furthermore show that DEM errors significantly increased with increasing slope value above 6°, which can be used to corrected the systematic errors caused by terrain slope angles [53]. However, no relationship was found between the vertical error and terrain aspect, whereas the DEM vertical accuracy significantly decreases as the land cover ranges from non-vegetated to vegetated.

Overall, ASTER GDEM2 performed worst, while SRTM1 DEM showed a very robust performance. Thus, SRTM1 is the most promising dataset with high accuracy and spatial resolution, and thus the future applications should utilize SRTM1 DEM. AW3D30 DEM, however, is another good choice for 1-arc-second resolution. The findings of this study are important to understand the error associated with these freely available DEMs. However, due to few ICESat-2 sampling data, it is difficult to unravel the influence of each factors (e.g., elevation, slope, land cover type) on DEM accuracy. In addition, multitemporal land cover type map is also crucially important to explore the influence of land cover changes on the results obtained. Therefore, further studies and analysis need to answer these questions.

## Appendix A

**Table A1:** Summary of the selected DEM dataset.

Data Sources	Provider	Method	Grid	Geographic Datum	Coordinate System	Coverage	Veritical Accuracy	Access Website
SRTM (v1, v2 v3, v4.1)	NASA	C-band InSAR	1″/3″	WGS84	EGM96	56° S~60° N	16 m (LE90)	https://earthexplorer.usgs.gov/
ASTER (v1, v2, v3)	METI NASA	ASTER stereopairs	1″	WGS84	EGM96	83° S~83° N	20 m (LE95)	https://earthexplorer.usgs.gov/
AW3D30 (v1.0, v1.1, v2.1, v2.2,v3.1)	JAXA	PRISM stereopairs	1″	WGS84	EGM96	80° S~80° N	3 m	https://www.eorc.jaxa.jp/ALOS/en/aw3d30/data/index.htm
TDX90	DLR	X-band InSAR	3″	WGS84	WGS84 (G1150)	90° S~90° N	10 m	https://geoservice.dlr.de/web/dataguide/tdm90/
ICESat-1	NASA	Full-waveform	170 m	TOPEX/Poseidon	TOPEX/Poseidon	90° S~90° N		https://search.earthdata.nasa.gov/search
ICESat-2	NASA	Photon-counting	70 cm	WGS84	WGS84	90° S~90° N	0.85 m	https://search.earthdata.nasa.gov/search

Note: More detail information can refer to [33,43,44,49,67,68,69].
